# Electroacupuncture Alleviates Neuroinflammation by Inhibiting the HMGB1 Signaling Pathway in Rats with Sepsis-Associated Encephalopathy

**DOI:** 10.3390/brainsci12121732

**Published:** 2022-12-17

**Authors:** Yueyang Xin, Jinxu Wang, Tiantian Chu, Yaqun Zhou, Cheng Liu, Aijun Xu

**Affiliations:** 1 Department of Anesthesiology, Tongji Hospital, Tongji Medical College, Huazhong University of Science and Technology, 1095 Jiefang Avenue, Wuhan 430030, China; 2 Department of Anesthesiology, Beijing Chao-Yang Hospital, Capital Medical University, Beijing 100020, China

**Keywords:** neuroinflammation, electroacupuncture, HMGB1, sepsis-associated encephalopathy

## Abstract

Sepsis-Associated Encephalopathy (SAE) is common in sepsis patients, with high mortality rates. It is believed that neuroinflammation is an important mechanism involved in SAE. High mobility group box 1 protein (HMGB1), as a late pro-inflammatory factor, is significantly increased during sepsis in different brain regions, including the hippocampus. HMGB1 causes neuroinflammation and cognitive impairment through direct binding to advanced glycation end products (RAGE) and Toll-like receptor 4 (TLR4). Electroacupuncture (EA) at Baihui (GV20) and Zusanli (ST36) is beneficial for neurological diseases and experimental sepsis. Our study used EA to treat SAE induced by lipopolysaccharide (LPS) in male Sprague–Dawley rats. The Y maze test was performed to assess working memory. Immunofluorescence (IF) and Western blotting (WB) were used to determine neuroinflammation and the HMGB1 signaling pathway. Results showed that EA could improve working memory impairment in rats with SAE. EA alleviated neuroinflammation by downregulating the hippocampus’s HMGB1/TLR4 and HMGB1/RAGE signaling, reducing the levels of pro-inflammatory factors, and relieving microglial and astrocyte activation. However, EA did not affect the tight junctions’ expression of the blood–brain barrier (BBB) in the hippocampus.

## 1. Introduction

Sepsis is a life-threatening organ dysfunction caused by a dysregulated host response to infection [1], which causes various complications, including cardiac disorders, liver and kidney dysfunction, and brain damage. Brain damage known as SAE occurs earlier and is more common [2], resulting in agitation, hallucinations, lack of focus, sleep–wake cycle disturbance, somnolence, and even coma [3]. Although the pathogenesis of SAE remains unclear, the pathophysiology is certainly multifactorial [4]. Neuroinflammation and blood–brain barrier (BBB) impairment are both involved in the pathogenesis of SAE [5]. Neuroinflammation is defined as an inflammatory response in the brain or spinal cord. Mediators, including cytokines, chemokines, reactive oxygen species, and second messengers produced by glia (microglia and astrocytes), endothelial cells, and immune cells infiltrated from the periphery to the central nervous system (CNS), all participate in neuroinflammation [6]. The neuroinflammatory response of sepsis is not due to cerebral infection, but systemic inflammation transmits to the innate immunity of the CNS through various pathways [7]. The BBB consists of vascular endothelial cells, pericytes, extracellular matrix, and endfeet of astrocytic processes. It is a barrier to the entry of microorganisms, toxins, bioactive substances, and a variety of substances, including drugs, into the brain [8]. Its disruption is often connected with neuroinflammation [9]. HMGB1 participates in the systemic inflammatory response as a late pro-inflammatory factor. It usually presents in the nucleus and binds to DNA. Under inflammatory conditions, it can be transferred from the nucleus to the cytoplasm and eventually released from the cell. HMGB1 can promote the production other inflammatory factors by activating the nuclear factor-κB (NF-κB) through binding to advanced glycation end products (RAGE) and Toll-like receptor 4 (TLR4) [10,11,12]. Current studies have demonstrated that HMGB1 plays an important role in inducing neuroinflammation and cognitive impairment in SAE [13,14]. Targeting HMGB1 signaling is expected to be a new therapeutic direction for ameliorating neuroinflammation in SAE.

It is reported that the incidence of SAE in patients with sepsis is about 70%, and the mortality rate in those is 56.1% [15]. However, no evidence-based treatment options are available for SAE patients, and non-drug treatments may be safer and more effective [3]. Hence, finding more viable non-drug treatments is beneficial for SAE.

According to traditional Chinese medicine (TCM), the theoretical basis of acupuncture includes the “qi” and “meridian”; “qi” refers to the vital energy in our body and flows through a network of channels called “meridians and collaterals”, which regulate yin and yang flow and keep our body in harmony. The disease occurs when the flow is interrupted [16,17,18]. Acupoints are the loci where the qi of zang-fu organs and meridians are transported to the body’s surface. Each point in a specific area can be used to treat any disorder in nearby tissues and organs [18]. Baihui (GV20) belongs to the Du meridian (the government vessel) and is technically used in neurological and psychiatric diseases because of its role in clearing the mind, lifting the spirits, promoting resuscitation, and so on [19]. Meanwhile, Zusanli (ST36) is located on the stomach meridian and is usually used to adjust the spleen and stomach and improve general weakness [20]. However, it has been proved that acupuncture at ST36 has an anti-inflammatory effect and can be used for treating experimental sepsis [21,22]. Unlike manual acupuncture (MA), which requires inserting a thin metal needle into an acupoint and manually operating it, electroacupuncture (EA) is a comparatively new method. Electronic instruments stimulate the acupoint and transmit electrical signals to achieve the purpose of treatment. Compared with MA, EA has better consistency and repeatability in scientific studies [23,24].Therefore, we hypothesized that EA at GV20 and ST36 could ameliorate neuroinflammation during SAE by modulating HMGB1 signaling.

## 2. Materials and Methods

### 2.1. Animals

A total of 60 male Sprague–Dawley rats (weight: 200–220 g, age: 6 weeks) were obtained from Tongji Hospital, Huazhong University of Science and Technology (Wuhan, China), and maintained at room temperature (22 ± 2 °C) under a standard 12–12 h light–dark cycle with constant humidity (55% ± 10%) in specific pathogen-free conditions for at least one week before beginning the experiment. Notably, in our experiment, only male rats were selected based on the following considerations: First, according to the demographics, men are the majority gender in patients with sepsis [25,26]. Second, numerous animal experiments reported that sex might influence the outcome of sepsis, and estrogen may have a protective effect on sepsis [27,28]. Hence, we only used male rats for our research to avoid the potential protective effect of estrogen on sepsis. Our study was approved by the Experimental Animal Care and Use Committee of Tongji Medical College, Huazhong University of Science and Technology (SYXK2019-0106). The committee’s reference number is TJH-202107005. All rats were allowed free access to the standard diet and tap water and were randomly divided into four groups: sham group (n = 15), EA group (n = 15), LPS group (n = 15), and EA+LPS group (n = 15). All procedures were implemented following the National Institutes of Health Guidelines for the Care and Use of Laboratory Animals.

### 2.2. Electroacupuncture Treatment

From the beginning of day 1, in the EA group and EA+LPS group, rats received EA for the following steps: First, rats were anesthetized through intraperitoneal injection of pentobarbital sodium 50 mg/kg. Then, the acupoints were selected as Baihui (GV20) and left Zusanli (ST36). The location of the above acupoints refers to the transpositional acupoint system in mouse and rat models [29]. Next, the acupoints were pierced by sterile acupuncture needles (0.25 × 25 mm, Hwato, Suzhou, China), and two electrodes from the Hwato Nerve and Muscle Stimulator (model no. SDZ-V, Suzhou, China) were attached to the end of acupuncture needles as reported previously [30,31]. The intensity was 1 mA, and the frequency was 15 Hz for 20 min. EA was conducted once daily until day 4. For the sham group and LPS group, rats were also anesthetized through intraperitoneal injection of pentobarbital sodium 50 mg/kg to reduce variables.

### 2.3. Sepsis-Associated Encephalopathy Model

**Figure 1:**
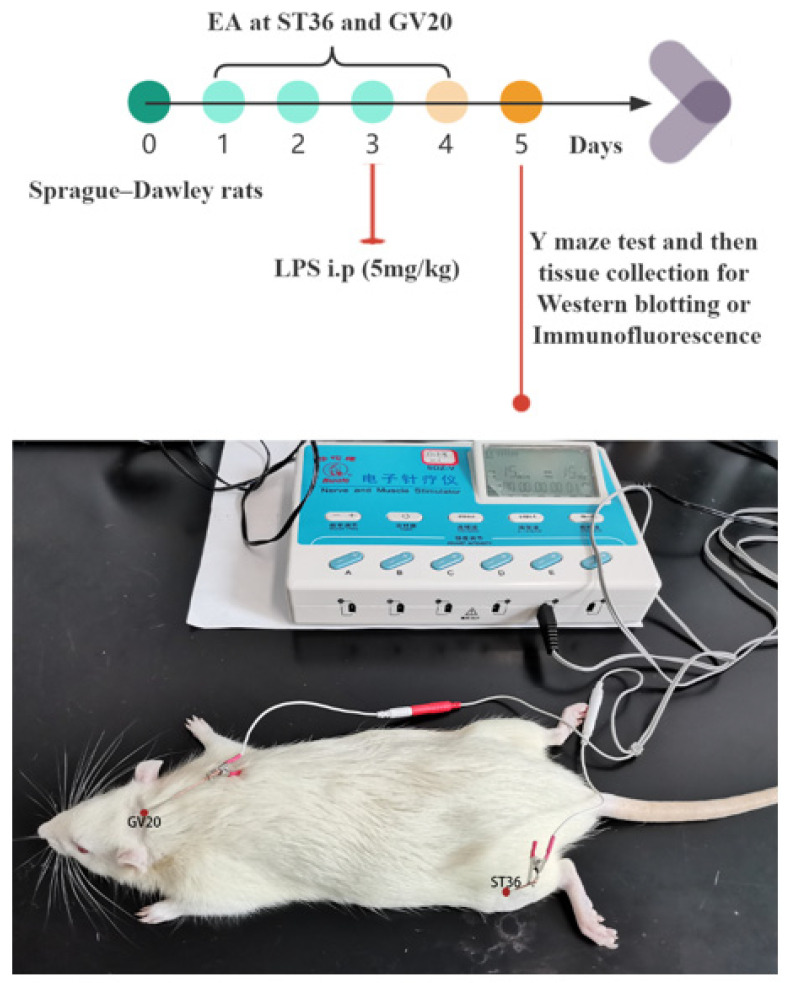
Experimental design and EA diagram. EA treatment was given at 15 Hz and an intensity of 1 mA for 20 min once a day from day 1 to day 4. Abbreviations: EA, electroacupuncture; LPS, lipopolysaccharide; GV20, Baihui; ST36, Zusanli; i.p., intraperitoneal injection.

For the LPS group and EA+LPS group, SAE was induced by intraperitoneal injection of LPS (E. coli, strain 055: B5, L2880, Sigma-Aldrich, Saint Louis, MO, USA) 5 mg/kg [32,33,34] on day 3. LPS was dissolved in normal saline (2 mg/mL) and the final volume was 2.5 mL/kg. Rats in other groups were intraperitoneally injected with an equal volume of normal saline on day 3. Our experimental design and EA diagram are presented in Figure 1.

### 2.4. Y Maze Test

Spatial working memory was measured using a Y maze (arm parameter: 500 × 100 × 300 mm, ZS-MGY, Zhongshi Technology, Beijing, China) test on day 5 in the evening, given the characteristics of the behavior of rats [35]. The angle between the arms is 120°. The Y maze test was performed as reported previously [36]. Rats were all naïve to the Y maze. Briefly, rats alternately explored three arms, which were artificially divided into A, B, C, and central areas. Rats were placed in the central area, and the orders in which they entered each arm were recorded within 8 min. Spontaneous alternation in rats refers to rodents’ natural tendency to choose alternate arms spontaneously. For example, ABC, ACB, BAC, BCA, CBA, CAB, etc., are correct alterations. The spontaneous alternation percentage (SAP) was calculated as follows: number of correct alterations/(total number of records − 2) × 100%. To eliminate the smell of the previous rat, we used 75% alcohol to clean the maze. The interval between the two rats was 5 min to guarantee that the 75% alcohol was volatilized entirely.

### 2.5. Western Blotting

The rats were sacrificed after the Y maze test under deep anesthesia on day 5. Then, the hippocampus was immediately removed and homogenized in an ice-cold mixture of RIPA lysis buffer, a phosphatase inhibitor, and a protease inhibitor (AR1183, Boster Biological Technology, Wuhan, China). Total protein was extracted on ice from supernatants after centrifugation at 12,000 rpm for 15 min. The protein concentration was detected using a BCA protein assay kit (AR0146, Boster Biological Technology, Wuhan, China). The proteins were boiled at 100 °C in the loading buffer for 10 min and stored at −80 °C until use. Samples (25 μg protein) were loaded and separated using 10% or 12% sodium dodecyl sulfate-polyacrylamide gel electrophoresis and then transferred to a polyvinylidene fluoride membrane. After blocking with 5% skim milk or 5% BSA in Tris-buffered saline and Tween 20 (TBST, 0.1%) at room temperature, the membranes were incubated at 4 °C overnight with primary antibodies: rabbit anti-HMGB1 antibody (A2553, 1:1000, Abclonal, Wuhan, China), rabbit anti-glial fibrillary acidic protein (GFAP) antibody (A0237, 1:1000, Abclonal, Wuhan, China), rabbit anti-Occludin antibody (A2601, 1:1000, Abclonal, Wuhan, China), rabbit anti-connexin 43 (Cx43) antibody (A11752, 1:2000, Abclonal, Wuhan, China), rabbit anti-TNF-α antibody (A0277, 1:1500, Abclonal, Wuhan, China), rabbit anti-ionized calcium-binding adapter molecule 1 (Iba1) antibody (A19776, 1:500, Abclonal, Wuhan, China), rabbit anti-zonula occludens-1 (ZO-1) antibody (AF5145, 1:1000, Affinity Biosciences, OH, USA), rabbit anti-TLR4 antibody (AF7017, 1:2000, Affinity Biosciences, OH, USA), rabbit anti-IL-1β antibody (A20529, 1:1000, Abclonal, Wuhan, China), rabbit anti-NF-κB antibody (A19653, 1:2000, Abclonal, Wuhan, China), rabbit anti-RAGE antibody (A13264, 1:1500, Abclonal, Wuhan, China), rabbit anti-Phospho-NF-κB p65 (Ser536) antibody (3033, 1:1000, Cell Signaling Technology, Boston, MA, USA), rabbit anti-IL-6 antibody (21865-1-AP, 1:1000, Proteintech, Wuhan, China), mouse anti-glyceraldehyde 3-phosphate dehydrogenase (GAPDH) antibody (10494-1-AP, 1:8000, Proteintech, Wuhan, China), and mouse anti-β-Actin monoclonal antibody (66009-1-lg, 1;8000, Proteintech, Wuhan, China). After washing in TBST three times for 10 min each, the membranes were incubated with horseradish peroxidase (HRP)-conjugated goat anti-mouse secondary antibody (AS003, 1:5000, Abclonal, Wuhan, China) and goat anti-rabbit secondary antibody (AS014, 1:5000, Abclonal, Wuhan, China) for 1.5 h at room temperature. Finally, the protein bands were measured by a SuperLumia ECL Plus HRP Substrate Kit (K22030, Abbkine, Wuhan, China) and exposed using a ChemiDoc XRS+ imaging system (Version:5.2.1, Bio-Rad, Hercules, CA, USA). The protein blots were analyzed using ImageJ (Version: 1.52, National Institutes of Health, Bethesda, MD, USA). Gray value was calculated by the “Gels” function under the “Analyze” tool. The gray value of the target protein was compared with that of the reference protein as the relative expression.

### 2.6. Immunofluorescence (IF)

Rats were sacrificed under deep anesthesia. Then, the brain was removed rapidly and fixed in 4% paraformaldehyde at 4 °C for 48 h. The rat brain was dehydrated in a gradient and coated with paraffin. Next, the brain was cut into 5 μm thick continuous coronal brain slices beginning 3 mm from the anterior tip of the frontal lobe. We selected the hippocampal plane from “Bregma: −5.16 mm, Interaural 3.84 mm” to “Bregma: −5.52 mm, Interaural 3.48 mm” according to *The Rat Brain in Stereotaxic Coordinates* [37]. To achieve a relatively small margin of error, we first stained the sections near the middle of our selected target area. Then, a morphological comparison was conducted according to *The Rat Brain in Stereotaxic Coordinates*. The variations in sections that were no more than two anteroposterior adjacent planes in *The Rat Brain in Stereotaxic Coordinates* were used for cell counting. Sections were processed by normal dewaxing and rehydrated. The sections were placed in 3% BSA for 30 min and then incubated with rabbit anti-GFAP antibody (ab7260, 1:5000, Abcam, Cambridge, UK) or rabbit anti-Iba1 antibody (ab178846, 1:2000, Abcam, Cambridge, UK) overnight at 4 °C. After being washed with 0.01M PBS three times, the sections were incubated with Cy3 Goat Anti-Rabbit IgG (H+L) (AS007, 1:200, Abclonal, Wuhan, China) or Goat Anti-Rabbit IgG H&L (Alexa Fluor^®^ 488, ab150077, 1:200, Abcam, Cambridge, UK) for 50 min at room temperature. Lastly, the sections were stained with 4′,6-diamidino-2-phenylindole (DAPI) (C0065, Servicebio, Wuhan, China) and observed under a fluorescence microscope (Olympus cellSens Standard, version: 3.2, Tokyo, Japan). For the analysis of GFAP-positive and Iba1-positive cells, three areas of fixed size (an area of 400 μm × 200 μm) in each section were randomly acquired in the dentate gyrus (DG) region and CA1 region by using Image J (Version: 1.52, National Institutes of Health, Bethesda, MD, USA). The 6 measurements (3 from the DG area and 3 from the CA1 area) from each rat were averaged to derive the value for the rat.

### 2.7. Statistical Analyses

The Shapiro–Wilk test was used to determine the normal distribution of continuous variables. When the continuous variables were normally distributed, they were analyzed by two-way ANOVA, followed by Bonferroni’s post hoc test to compare with every other group, and presented as the mean ± standard error of the mean (SEM), such as the results of Western blot and SAP. When the continuous variables were not normally distributed, they were subjected to the Kruskal–Wallis test followed by Dunn’s post hoc test for nonparametric statistical analysis to compare with every other group, such as the total number of records in the Y maze was presented as the median and interquartile range. *p* values less than 0.05 were considered statistically significant. We used GraphPad Prism 9 (GraphPad Software, version: 9.4.0, San Diego, CA, USA) to complete data statistics and analysis.

## 3. Results

### 3.1. EA Mitigated Working Memory Impairment Caused by LPS

**Figure 2:**
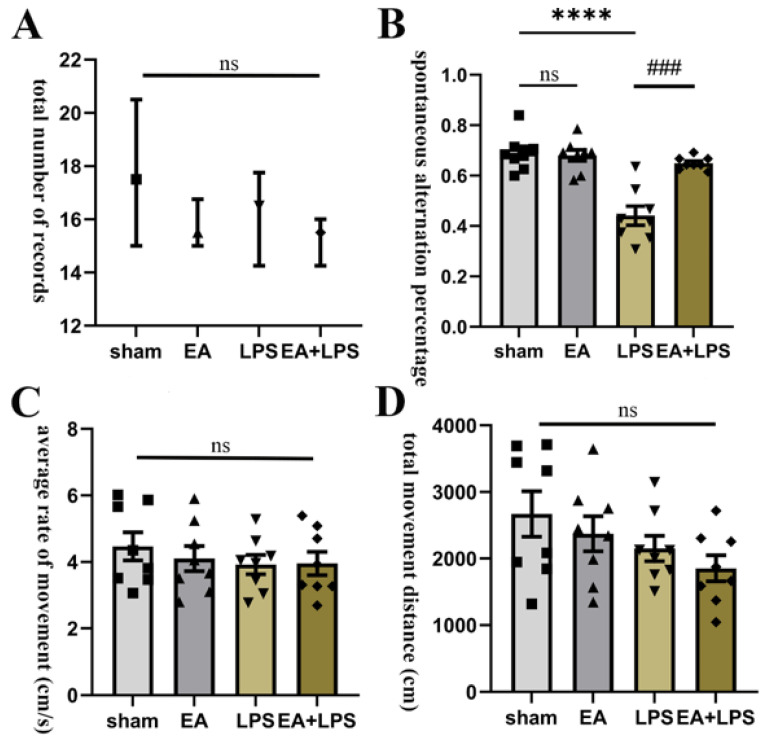
EA improved working memory tested by Y maze: n = 8 per group. (**A**) The total number of records in each group. (**B**) The spontaneous alternation percentage in each group. (**C**) The average rate of movement in each group. (**D**) The total movement distance in each group. Data are presented as median and interquartile range and means ± SEM. Compared with the sham group, **** *p* < 0.0001; compared with the LPS group, ^###^ *p* < 0.001. ■, ▲, ▼, ◆: Represents the individual value of the rats in each group. Abbreviations: EA, electroacupuncture; LPS, lipopolysaccharide; ns, no significant difference.

The Y maze test was used to evaluate EA’s effects on spatial working memory. First, we analyzed the number of records in each group. As shown in Figure 2A, there was no significant difference in the number of records among all groups (*p* > 0.05), and there were no significant differences in the average rate of movement and the total movement distance among the four groups (Figure 2C,D, *p* > 0.05). Then, we analyzed the SAP continuously by one-way ANOVA and Bonferroni’s post hoc test. The results indicated that rats revealed significant cognitive impairment in the LPS group compared with the sham group (*p <* 0.0001, Figure 2B). Moreover, the impairment was mitigated significantly in the EA+LPS group compared to the LPS group (*p <* 0.001, Figure 2B). There was no statistical difference between sham and EA groups (*p* > 0.999). These results demonstrated that EA could improve working memory in LPS-induced SAE rats.

### 3.2. EA Modulated the HMGB1 Signaling in the Hippocampus

**Figure 3:**
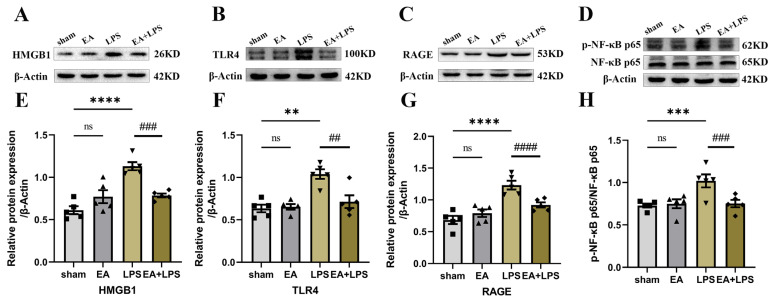
EA modulated HMGB1 signaling in the hippocampus: n = 5 per group. (**A**) Representative blots of HMGB1. (**B**) Representative blots of TLR4. (**C**) Representative blots of RAGE. (**D**) Representative blots of p-NF-κB p65 and NF-κB p65. (**E**) Quantification of the expression level of HMGB1. (**F**) Quantification of the expression level of TLR4. (**G**) Quantification of the expression level of RAGE. (**H**) Quantification of the ratio of p-NF-κB p65 and NF-κB p65. Data are presented as means ± SEM. Compared with the sham group, ** *p* < 0.01, *** *p* < 0.001, and **** *p* < 0.0001; compared with the LPS group, ^##^ *p* < 0.01, ^###^ *p <* 0.001, and ^####^ *p <* 0.0001. ■, ▲, ▼, ◆: Represents the individual value of the rats in each group. Abbreviations: HMGB1, high mobility group box 1 protein; NF-κB, nuclear factor-κB; p- NF-κB, phosphorylated-NF-κB; RAGE, the receptor for advanced glycation end products; TLR4, Toll-like receptor 4; EA, electroacupuncture; LPS: lipopolysaccharide; ns: no significant difference.

Changes in HMGB1 signaling were detected by Western blotting. As demonstrated in Figure 3, the expression levels of HMGB1 (Figure 3A,E, *p <* 0.0001), TLR4 (Figure 3B,F, *p <* 0.01), and RAGE (Figure 3C,G, *p <* 0.0001) and the ratio of phosphorylated-NF-κB p65 (p-NF-κB p65)/NF-κB p65 (Figure 3D,H, *p <* 0.001) were significantly increased in the LPS group compared to those in the sham group. Nevertheless, the variations above were all alleviated significantly by EA (Figure 3E–H, for HMGB1 and the ratio of p-NF-κB p65/NF-κB p65, *p <* 0.001, for TLR4, *p <* 0.01, for RAGE, *p <* 0.0001). In the meantime, there were no significant differences between the sham group and EA group (for HMGB1, *p* = 0.1601, for RAGE, *p* = 0.0859, for TLR4 and the ratio of p-NF-κB p65/NF-κB p65, *p* > 0.999). Based on these data, our research indicated that EA could decrease the activation of HMGB1 signaling in the hippocampus of rats with SAE.

### 3.3. EA Alleviated Neuroinflammation in the Hippocampus

**Figure 4:**
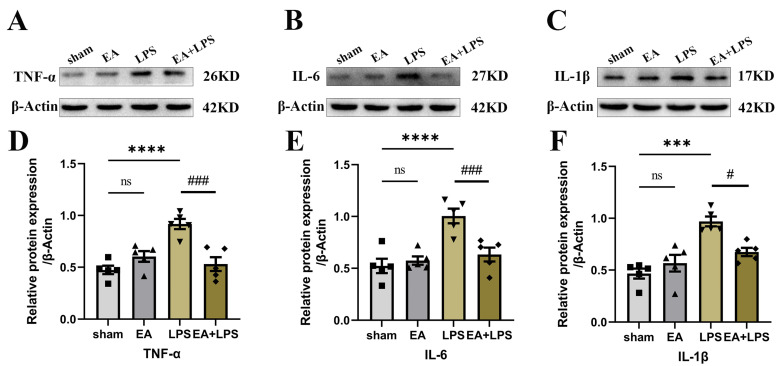
EA decreased pro-inflammatory cytokines in the hippocampus: n = 5 per group. (**A**) Representative blots of TNF-α. (**B**) Representative blots of IL-6. (**C**) Representative blots of IL-1β. (**D**) Quantification of the expression level of TNF-α. (**E**) Quantification of the expression level of IL-6. (**F**) Quantification of the expression level of IL-1β. Data are presented as means ± SEM. Compared with the sham group, *** *p* < 0.001, **** *p* < 0.0001; compared with the LPS group, ^#^ *p* < 0.05, ^###^ *p* < 0.001. ■, ▲, ▼, ◆: Represents the individual value of the rats in each group. Abbreviations: IL-1β, interleukin-1β; IL-6, interleukin-6; TNF-α, tumor necrosis factor α; EA: electroacupuncture; LPS, lipopolysaccharide; ns, no significant difference.

**Figure 5:**
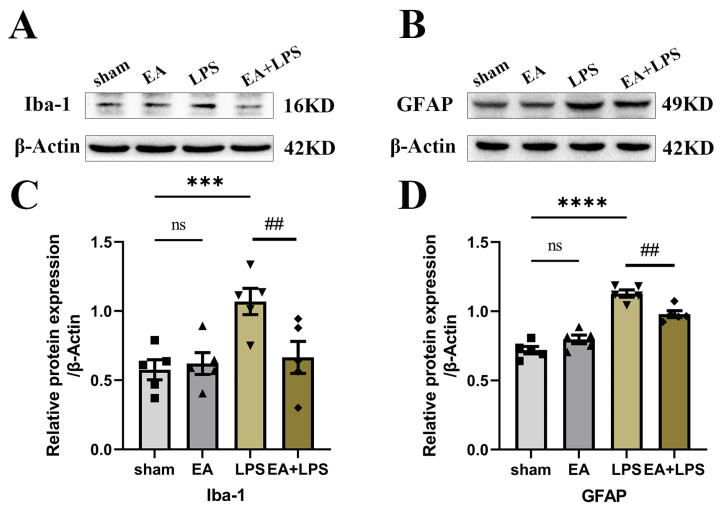
EA suppressed the expression level of Iba-1 and GFAP in the hippocampus: n = 5 per group. (**A**) Representative blots of Iba-1. (**B**) Representative blots of GFAP. (**C**) Quantification of the expression level of Iba-1. (**D**) Quantification of the expression level of GFAP. Data are presented as means ± SEM. Compared with the sham group, *** *p* < 0.001, **** *p* < 0.0001; compared with the LPS group, ^##^ *p* < 0.01. ■, ▲, ▼, ◆: Represents the individual value of the rats in each group. Abbreviations: GFAP, glial fibrillary acidic protein; Iba-1, ionized calcium-binding adapter molecule 1; EA, electroacupuncture; LPS, lipopolysaccharide; ns, no significant difference.

**Figure 6:**
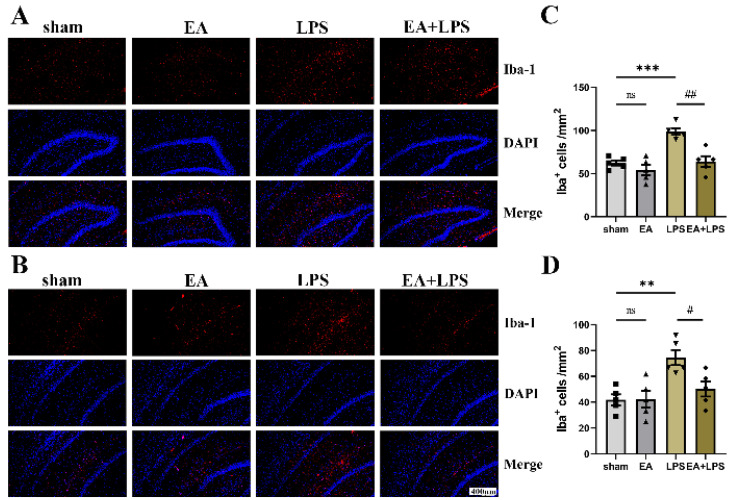
EA alleviated the activation of microglia in the DG and CA1 regions, n = 5 per group. (**A**) Representative IF images of activated microglia in the DG region. (**B**) Representative IF images of activated microglia in the CA1 region. (**C**) The statistical results of Iba-1^+^ cell count in the DG region. (**D**) The statistical results of Iba-1^+^ cell count in the CA1 region. Magnification: 20×, scale bar = 400 µm. Data are presented as means ± SEM. Compared with the control group, ** *p* < 0.01, *** *p* < 0.001; compared with the LPS group, ^##^ *p* < 0.01, ^#^ *p* < 0.05. ■, ▲, ▼, ◆: Represents the individual value of the rats in each group. Abbreviations: DAPI, 4′,6-diamidino-2-phenylindole; DG, dentate gyrus; Iba-1, ionized calcium-binding adapter molecule 1; EA, electroacupuncture; LPS, lipopolysaccharide; ns, no significant difference.

**Figure 7:**
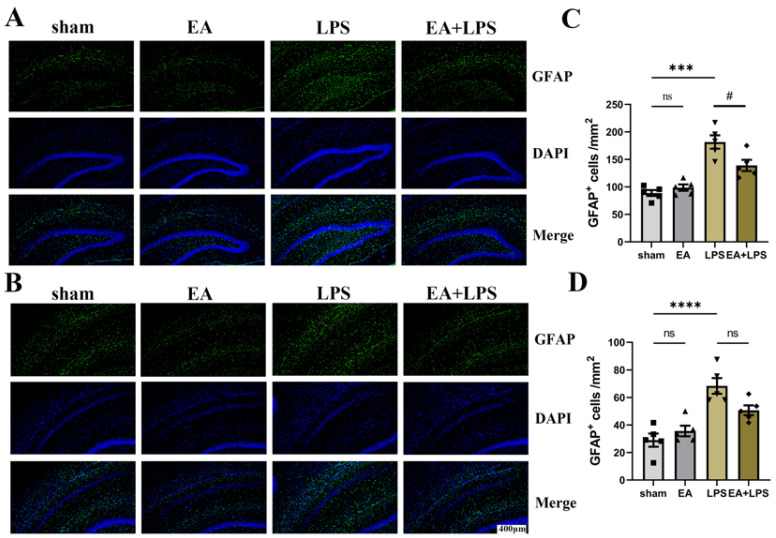
EA affected the activation of astrocytes in the DG and CA1 region: n = 5 per group. (**A**) Representative IF images of activated astrocytes in the DG region. (**B**) Representative IF images of activated astrocytes in the CA1 region. (**C**) The statistical results of GFAP^+^ cell count in the DG region. (**D**) The statistical results of GFAP^+^ cell count in the CA1 region. Magnification: 20×, scale bar = 400 µm. Data are presented as means ± SEM. Compared with the control group, *** *p* < 0.001, **** *p* < 0.0001; compared with the LPS group, ^#^ *p* < 0.05. ■, ▲, ▼, ◆: Represents the individual value of the rats in each group. Abbreviations: DAPI, 4′,6-diamidino-2-phenylindole; DG, dentate gyrus; GFAP, glial fibrillary acidic protein; EA, electroacupuncture; LPS, lipopolysaccharide; ns, no significant difference.

To investigate the effects of EA on neuroinflammation induced by SAE, we detected the expression levels of TNF-α, IL-6, and IL-1β in the hippocampus using Western blotting. Compared with sham group, rats in the LPS group possessed higher contents of TNF-α (Figure 4A,D, *p* < 0.0001), IL-6 (Figure 4B,E, *p* < 0.0001), and IL-1β (Figure 4C,F, *p* < 0.001) in the hippocampus. However, in the EA+LPS group, compared with the LPS group, the expression levels of TNF-α (Figure 4A,D, *p* < 0.001), IL-6 (Figure 4B,E, *p* < 0.001), and IL-1β (Figure 4C,F, *p* < 0.05) were significantly reduced after EA treatment. In addition, no significant difference existed between sham and EA groups (for TNF-α, *p* = 0.3742, for IL-6 and IL-1β, *p* > 0.999). Then, we conducted Western blotting and IF in the hippocampus to assess whether EA affected microglial and astrocyte activation. The results showed that the expression level of Iba-1 (Figure 5A,C, *p* < 0.001) and GFAP (Figure 5B,D, *p* < 0.0001) was significantly elevated in the LPS group compared with the sham group. After treatment by EA, the expression levels of Iba-1 (Figure 5A,C, *p* < 0.01) and GFAP (Figure 5B,D, *p* < 0.01) were significantly lower compared with the LPS group. Similarly, when compared with the sham group, there was no significant difference in the EA group (for GFAP, *p* = 0.2724, for Iba-1, *p* > 0.999). Next, we counted the number of Iba-1^+^ and GFAP^+^ cells in DG and CA1 areas. In the LPS group, both Iba-1^+^ and GFAP^+^ cells in DG and CA1 areas were statistically increased when compared with the sham group (Figure 6A,C, *p* < 0.001, Figure 6B,D, *p* < 0.01, Figure 7A,C, *p* < 0.001, Figure 7B,D, *p* < 0.0001). EA could significantly reduce Iba-1^+^ cells in these areas (Figure 6A,C, *p* < 0.01, Figure 6B,D, *p* < 0.05). However, EA only significantly reduced GFAP^+^ cells in the DG area rather than in CA1 (Figure 7A,C, *p* < 0.05, Figure 7B,D, *p* = 0.0664). For Iba-1^+^ cells and GFAP^+^ cells, the results showed no significant difference between the sham group and EA group (*p* > 0.999). In summary, EA alleviated neuroinflammation in the hippocampus by reducing the contents of TNF-α, IL-6, and IL-1β, as well as relieving the activation of microglia and astrocyte in rats with SAE.

### 3.4. EA Did Not Affect the Tight Junctions’ Expression of BBB in the Hippocampus

**Figure 8:**
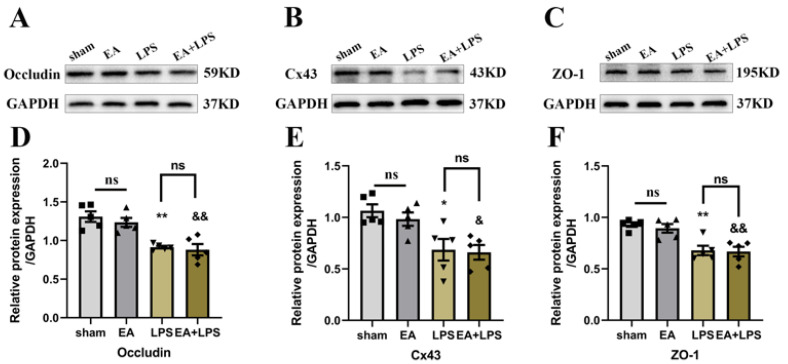
EA was ineffective in the junction proteins of the BBB: n = 5 per group. (**A**) Representative blots of Occludin. (**B**) Representative blots of Cx43. (**C**) Representative blots of ZO-1. (**D**) Quantification of the expression level of Occludin. (**E**) Quantification of the expression level of Cx43. (**F**) Quantification of the expression level of ZO-1. Data are presented as means ± SEM. Compared with the sham group, ** *p* < 0.01, * *p* < 0.05, && *p* < 0.01, & *p* < 0.05. ■, ▲, ▼, ◆: Represents the individual value of the rats in each group. Abbreviations: Cx43, connexin 43; ZO-1, zonula occludens-1; GAPDH, glyceraldehyde 3-phosphate dehydrogenase; EA, electroacupuncture; LPS, lipopolysaccharide; ns, no significant difference.

ZO-1 and Occludin are essential components of tight junctions, and Cx43 is one of the gap junctions [38,39]. Therefore, we detected the expression levels of Occludin, Cx43, and ZO-1 in the hippocampus using Western blotting to evaluate BBB dysfunction. In the LPS group, the contents of Occludin (Figure 8A,D, *p* < 0.01), Cx43 (Figure 8B,E, *p* < 0.05), and ZO-1 (Figure 8C,F, *p* < 0.01) were significantly lower than those in sham group. However, the expression levels of Occludin (Figure 8A,D, *p* > 0.999), Cx43 (Figure 8B,E, *p* > 0.999), and ZO-1 (Figure 8C,F, *p* > 0.999) in the EA+LPS group were not significantly improved compared with the LPS group. In the EA group, there were no significant differences in tight junctions’ expression compared with the sham group (*p* > 0.999). These results suggested that EA was ineffective in improving BBB dysfunction induced by SAE.

## 4. Discussion

In the present investigation, the role of HMGB1 signaling in rats with SAE was studied preliminarily. EA at GV20 and ST36 could significantly ameliorate HMGB1 signaling and neuroinflammation in LPS-induced SAE. Meanwhile, LPS-caused working memory impairment was diminished by EA.

The hippocampus, which can serve for memory by encoding all dimensions of experience, is all over the cognitive map [40]. In the acute stage of sepsis, hippocampus atrophy on brain magnetic resonance imaging (MRI) was shown, which was related to the SAE. Moreover, even the SAE survivors’ hippocampus volume was smaller than that of healthy controls [41]. Similarly, patients who suffered from sepsis-induced brain dysfunction (SIBD) had noticeable volume reduction in the hippocampus assessed by MRI [42]. Therefore, our research mainly focused on changes in HMGB1 signaling in the hippocampus. HMGB1 levels can be elevated in plasma and the hippocampus by intraperitoneal injection of LPS [43,44]. Once HMGB1 is released extracellularly through cell death or active cell secretion, it acts as a potent inflammatory mediator [45]. In murine models of endotoxemia induced by intraperitoneal injection of LPS, HMGB1 reaches plateau levels from 16 to 32 h [46,47]. The receptor for RAGE and TLR4, classified as pattern recognition receptors (PRR), can recognize several types of damage-associated molecular patterns (DAMP) to induce immune responses [48,49]. RAGE and TLR4 operate as the primary HMGB1 receptors, and HMGB1 acts as a pro-inflammatory mediator through direct binding to RAGE and TLR4 [50,51]. HMGB1/TLR4 and HMGB1/RAGE produce pro-inflammatory molecules by initiating the nuclear factor-κB (NF-κB) pathway [49,52,53]. Further production of inflammatory factors promotes the maturation and release of HMGB1, thus continuing to expand the effect of HMGB1 [12,54]. Previous reports showed that HMGB1 was significantly increased during sepsis in different brain regions, including the hippocampus [43,55]. In the meantime, it is widely accepted that HMGB1 release mediates hippocampal inflammation and contributes to cognitive impairment in preclinical models [56,57,58,59]. Intervention targeting the HMGB1/TLR4/NF-κB pathway could alleviate neuroinflammation and improve cognitive impairment in models of depression, cognitive impairment caused by high-fat and high-sugar diets, and traumatic brain injury [57,60,61,62]. Blocking HMGB1/RAGE signaling by Berberine also alleviates SAE’s cognitive deficits [63]. Similar to the above studies, our data proved that EA at GV20 and ST36 exerted protective effects by targeting HMGB1 signaling in the hippocampus.

In addition to HMGB1, activated microglia have recently been shown to promote SAE and are closely associated with cognitive changes [5]. When activated, microglia can generate many cytokines and metalloproteinases and cause neuronal dysfunction and memory impairment [5,64]. Activated microglia are consistently observed in both experimental models and septic patients; in particular, there is increased microglial activation in hippocampal tissue in patients who die of SAE [65,66,67]. In addition to microglial activation, astrocyte activation is also one of the most relevant phenomena in SAE [63,68,69]. It facilitates brain injury by releasing pro-inflammatory cytokines and toxic molecules related to neuroinflammation and is related to the severity of SAE [70,71]. The DG area has important cognitive roles and is critical to hippocampal function. In addition, the CAI region is associated with spatial novelty detection and was susceptible to behaviorally relevant and irrelevant changes [72,73]. The CAI region can encode new events into existing memory traces and discriminate between old and new stimuli [73,74]. In our research, we suggested that EA at GV20 and ST36 was an effective measure to reduce the expression levels of Iba-1 and GFAP in the whole hippocampus. Moreover, EA could mitigate the activation of microglia both in the DG region and CA1 region. EA decreased the activation level only in the DG region for the astrocytes. As for the CAI region, EA slightly reduced the GFAP^+^ cells. More studies are needed to further elucidate the effects of EA on different areas of the hippocampus in SAE patients.

BBB dysfunction is another factor involved in SAE etiology [75]. The function of the BBB to maintain CNS homeostasis is determined by its ability to control transport modes, rates, and regulation of ions, small molecules, immune cells, cytokines, chemokines, and exogenous compounds [76]. These functions cannot be achieved without endothelial cells, which form the core component of the BBB, including continuous intercellular tight junctions, low rates of transcytosis, and lack of fenestrations [77]. A large number of pro-inflammatory factors, such as TNF-α, IL-1β, and IL-6, and endotoxins can act on the BBB and then increase permeability in the process of sepsis. As a result, immune cells, inflammatory factors, and other substances from the periphery will enter the brain and induce dysfunction [4,5,34]. Notably, through active transport via specific carriers, cytokines can cross the BBB. However, in this way, only about 1% of circulating cytokines enter the brain [3]. TNF-α is an essential mediator of SAE because of its direct relationship with BBB dysfunction, brain edema, neutrophil infiltration, astrocytosis, and brain cell apoptosis [78,79]. The content of TNF-α in the hippocampus increased after LPS insult by intraperitoneal injection [80,81,82]. TNF-α and IL-1β following sepsis have also been considered as the critical factors causing cognitive impairment [83]. IL-6 also affects cognitive function in various diseases, including AD, Lewy body dementia, vascular dementia, cardiovascular disease, etc. [84]. Our study indicated that EA was a valuable method to lower the levels of inflammatory factors in the hippocampus, which may be partially involved in the EA mitigation of SAE. The effects of cytokines also include tight junctions’ impairment of the BBB [85]. Several preclinical studies have shown that targeting tight junctions is a helpful way to alleviate brain injury and improve cognitive impairment in SAE [67,86,87,88,89]. For intracerebral hemorrhage [90], cerebral ischemia/reperfusion [91], and cecal ligation and puncture models of rats [92], EA attenuates BBB disruption and decreases the contents of inflammatory cytokines. Nonetheless, Zhang et al. suggested that EA stimulation at a specific frequency could effectively enhance BBB permeability in rats [93]. In the present study, we preliminarily evaluated the tight junctions of the BBB. We observed that intraperitoneal injection of LPS reduced the expression levels of ZO-1, Occludin, and Cx43 in the hippocampus. However, EA did not affect BBB damage. In addition to the differences in animal models and the acupoints selection, therapeutic parameters of EA may also affect the final results. In addition, we only detected the tight junctions of the BBB, and other biomarkers of BBB permeability, such as ion channels and receptors, need further study. Therefore, more research is required to confirm the exact effects of EA on BBB integrity.

EA and MA serve as nonpharmacological and noninvasive approaches that have attracted the attention of the clinical medicine community [94]. Recent studies have shown that EA improves cognitive function in septic rats and intestinal function in septic patients [95,96,97]. A growing body of research indicates that EA can ameliorate neuroinflammation in animal models [30,98,99,100,101,102,103], which indicates good development prospects. Nonetheless, more research is still needed to evaluate and quantify the specific impact of EA on SAE.

Although this study initially demonstrated the therapeutic effect of EA on neuroinflammation after SAE by modulating the HMGB1 signaling pathway, elucidating the mechanism is still lacking in depth. Moreover, in the present study, we mainly focused on neuroinflammation in the hippocampus. The Y maze just assessed the working memory impairment. More behavioral experiments are needed to assess comprehensively whether cognitive function improves due to EA. Finally, we have mentioned that gender has an impact on the prognosis of sepsis. Therefore, the experimental results must be further verified on female animals before scientific extrapolation can be carried out.

## 5. Conclusions

**Figure 9:**
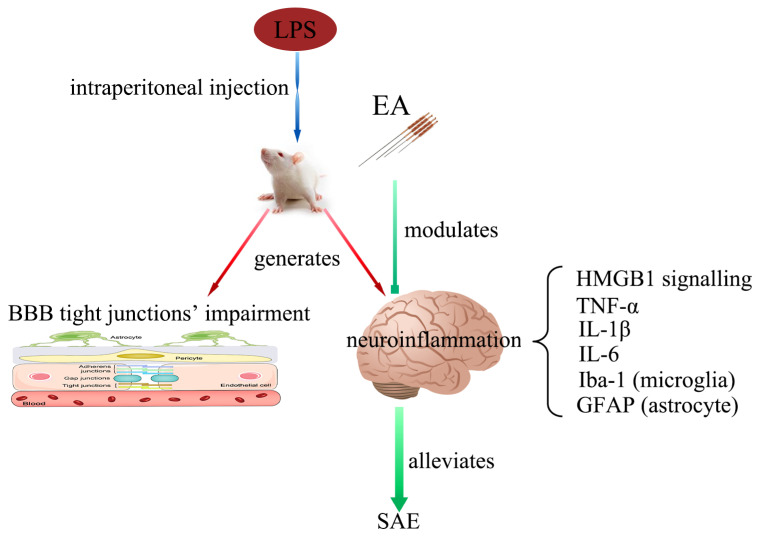
Working model: intraperitoneal injection of LPS to induce SAE impairs BBB’s tight junctions and evokes neuroinflammation in the hippocampus. The expression levels of HMGB1 signaling are enhanced, microglia and astrocytes are activated, and the content of pro-inflammatory factors is elevated. EA can modulate the HMGB1 signaling and relieve the intensity of neuroinflammation in the hippocampus. However, EA does not affect the tight junctions’ impairment of BBB. Abbreviations: BBB, blood–brain barrier; EA, electroacupuncture; GFAP, glial fibrillary acidic protein; HMGB1, high mobility group box 1 protein; Iba-1, ionized calcium-binding adapter molecule 1; IL-1β, interleukin-1β; IL-6, interleukin-6; LPS, lipopolysaccharide; TNF-α, tumor necrosis factor α; SAE: sepsis-associated encephalopathy.

In summary, in our present study, we demonstrate that LPS-induced SAE impairs the working memory of rats, activates the HMGB1/RAGE and HMGB1/TLR4 signaling in the hippocampus, increases the content of pro-inflammatory factors, and activates microglia and astrocytes in the hippocampus, which results in neuroinflammation. The BBB’s tight junctions were also damaged as well. According to our data, we suggest that EA exerts the protective effect of improving working memory and ameliorating neuroinflammation by inhibiting the HMGB1/RAGE and HMGB1/TLR4 signaling, reducing the expression level of the pro-inflammatory factors, and alleviating the activation of microglia and astrocytes in the hippocampus, rather than improving the damage of BBB’s tight junctions (Figure 9). Since only male SD rats were selected in our study, further studies with female animals are needed to confirm our conclusions.

## Data Availability

Information about the experimental methods, animal model, and data used and analyzed during the current study is available from the corresponding author upon reasonable request.
